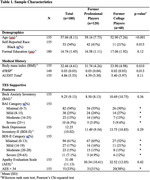# CSF Biomarkers and Supportive Psychiatric Features of the TES Criteria in former American Football Players

**DOI:** 10.1002/alz.091449

**Published:** 2025-01-09

**Authors:** Annalise E. Miner, Jenna Rae Groh, Yorghos Tripodis, Charles Adler, Laura Balcer, Charles B. Bernick, Henrik Zetterberg, Kaj Blennow, Nicholas J. Ashton, Elaine R. Peskind, Sarah Banks, William B. Barr, Jennifer V. Wethe, Robert C. Cantu, David W. Dodick, Yonas E. Geda, Douglas I Katz, Jason Weller, Jesse Mez, Joseph N. Palmisano, Brett Martin, Jeffrey L. Cummings, Eric M. Reiman, Martha E. Shenton, Robert A. Stern, Michael L. Alosco

**Affiliations:** ^1^ Boston University Chobanian & Avedisian School of Medicine, Boston, MA USA; ^2^ Boston University CTE Center, Boston University Chobanian & Avedisian School of Medicine, Boston, MA USA; ^3^ Boston University School of Public Health, Boston, MA USA; ^4^ Mayo Clinic, Scottsdale, AZ USA; ^5^ NYU Langone Health, New York City, NY USA; ^6^ NYU Grossman School of Medicine, New York, NY USA; ^7^ Lou Ruvo Center for Brain Health, Cleveland Clinic, Las Vegas, NV USA; ^8^ Department of Psychiatry and Neurochemistry, Institute of Neuroscience and Physiology, The Sahlgrenska Academy, University of Gothenburg, Mölndal, Gothenburg Sweden; ^9^ Department of Psychiatry and Neurochemistry, Institute of Neuroscience and Physiology, The Sahlgrenska Academy, University of Gothenburg, Mölndal Sweden; ^10^ VA Puget Sound Health Care System, Seattle, WA USA; ^11^ University of California, San Diego, La Jolla, CA USA; ^12^ Boston University Alzheimer’s Disease Research Center, Boston, MA USA; ^13^ Mayo Scottsdale, Scottsdale, AZ USA; ^14^ Mayo Clinic, Rochester, MN USA; ^15^ Boston University, Boston, MA USA; ^16^ Boston University Chronic Traumatic Encephalopathy Center, Boston University Chobanian & Avedisian School of Medicine, Boston, MA USA; ^17^ Cleveland Clinic Lou Ruvo Center for Brain Health, Las Vegas, NV USA; ^18^ Banner Alzheimer's Institute, Phoenix, AZ USA; ^19^ Brigham and Women's Hospital, Boston, MA USA; ^20^ Boston University Alzheimer’s Disease Research Center, Boston University Chobanian & Avedisian School of Medicine, Boston, MA USA

## Abstract

**Background:**

Traumatic encephalopathy syndrome (TES) is the proposed clinical syndrome of the neurodegenerative disease chronic traumatic encephalopathy (CTE). As part of the 2021 TES NINDS consensus diagnostic criteria, certainty levels of underlying CTE neuropathology can be determined (i.e., suggestive, possible, probable) partially based on the presence/absence of supportive psychiatric features including but not limited to depression, anxiety, and apathy. These are ‘supportive’ because of their non‐specificity and unclear biological correlates in this setting. We examined the association between CSF biomarkers of amyloid, tau, neurodegeneration, microglial activation and neuroinflammation and supportive psychiatric features of TES in former American football players.

**Method:**

The sample included 180 male former football players (120 professional, 60 college) from the DIAGNOSE CTE Research Project. Participants completed a lumbar puncture, and CSF assays were conducted for biomarkers of amyloid (Aβ40, Aβ42), p‐tau (p‐tau181, p‐tau231, p‐tau217), neurodegeneration (total tau), axonal degeneration (NfL), microglial activation (strem2) and neuroinflammation (GFAP, IL‐6). Biomarkers were log‐transformed. The Apathy Evaluation Scale (AES), Beck Depression Inventory‐II (BDI‐II) and the Beck Anxiety Inventory (BAI) self‐report questionnaires were completed by participants. Multivariable regression models tested the association between each CSF biomarker and each scale, controlling for age, race, body mass index, revised Framingham stroke risk profile and Alcohol Use Disorders Identification Test. P‐values were adjusted for multiple comparisons using false discovery rate methods.

**Result:**

140 participants had successful LPs and available CSF for assays. 33(22%) reported moderate/severe anxiety, 34(22%) reported moderate/severe depression, and 51(33%) had an elevated AES score. Higher CSF concentrations of IL‐6 were associated with higher scores on the BAI (unstandardized‐β=0.016, 95%CI [0.017, 0.026], p_adj_=0.004), BDI‐II (unstandardized‐β=0.013, 95%CI [0.004, 0.021], p_adj_=0.008) and AES (unstandardized‐β=0.014, 95%CI [0.006,0.022], p_adj_=0.002). There were no significant relationships between other biomarkers with the BDI‐II, BAI or AES.

**Conclusion:**

In this sample of former American football players, there were significant associations between IL‐6 (a proinflammatory cytokine) and reported symptoms of depression, anxiety, and apathy. CSF biomarkers of amyloid, tau and neurodegeneration had no associations, highlighting the role of neuroinflammatory processes in the manifestation of psychiatric symptoms of TES. Continued research on the biological correlates of TES features will inform future iterations of the criteria.